# Health Information Systems in the Digital Health Ecosystem—Problems and Solutions for Ethics, Trust and Privacy

**DOI:** 10.3390/ijerph17093006

**Published:** 2020-04-26

**Authors:** Pekka Ruotsalainen, Bernd Blobel

**Affiliations:** 1 Faculty for Information Technology and Communication Sciences, Tampere University, 33100 Tampere, Finland; 2 Medical Faculty, University of Regensburg, 93053 Regensburg, Germany; 3 Fist Medical Faculty, Charles University Prague, 12800 Prague, Czech Republic; 4 eHealth Competence Center Bavaria, Deggendorf Institute of Technology, 94469 Deggendorf, Germany

**Keywords:** ethics, privacy, trust, models, ethical design, computational privacy, fuzzy logic

## Abstract

Digital health information systems (DHIS) are increasingly members of ecosystems, collecting, using and sharing a huge amount of personal health information (PHI), frequently without control and authorization through the data subject. From the data subject’s perspective, there is frequently no guarantee and therefore no trust that PHI is processed ethically in Digital Health Ecosystems. This results in new ethical, privacy and trust challenges to be solved. The authors’ objective is to find a combination of ethical principles, privacy and trust models, together enabling design, implementation of DHIS acting ethically, being trustworthy, and supporting the user’s privacy needs. Research published in journals, conference proceedings, and standards documents is analyzed from the viewpoint of ethics, privacy and trust. In that context, systems theory and systems engineering approaches together with heuristic analysis are deployed. The ethical model proposed is a combination of consequentialism, professional medical ethics and utilitarianism. Privacy enforcement can be facilitated by defining it as health information specific contextual intellectual property right, where a service user can express their own privacy needs using computer-understandable policies. Thereby, privacy as a dynamic, indeterminate concept, and computational trust, deploys linguistic values and fuzzy mathematics. The proposed solution, combining ethical principles, privacy as intellectual property and computational trust models, shows a new way to achieve ethically acceptable, trustworthy and privacy-enabling DHIS and Digital Health Ecosystems.

## 1. Introduction

The Digital Era evolution started about 30 years ago and continues at increasing speed. This development has created global ecosystems characterized by ubiquitous use of digital technology such as computers, networks, platforms, clouds, algorithms and machine learning everywhere in society and business. They increasingly see personal information such as personal health information (PHI) as “new oil”, and collect, use and share it without limitations. This development has transformed the way health and health care services are provided and consumed. Thereby, new digital service models have been created such as eHealth (electronic health), pHealth (personalized health), mHealth (mobile health) and pervasive Health [1]. In the Digital Health Ecosystem, health care and health information systems are highly dynamic and fully distributed. Thereby, health information is dynamically collected, used and distributed between its members (stakeholders). Their sizes vary from a single application where PHI is collected by sensors, moved to a cloud, processed by an algorithm, and results are displayed on user’s mobile phone on one end, up to large digital health information systems (DHIS) on digital platforms and communication networks, using machine learning and artificial intelligence on the other end [2]. The services those digital health information systems provide are increasingly personalized, preventive and predictive, declared as 3P medicine, frequently extended by participative and precision medicine towards 5P medicine [3,4]. Both regulated and non-regulated health service providers can be members of the Digital Health Ecosystem and offer health services which take place outside the regulated health care domain [1]. To support personalized, preventive and predictive health, DHIS require a large amount of PHI, also needed for better understanding causes of diseases [5]. This all creates new ethical, privacy and trust challenges to be solved [6]. Currently, in Digital Health Ecosystems it is difficult or even impossible for a person or specifically for a patient to know what PHI is collected, used and disclosed by whom for what primary and secondary purposes. Furthermore, it is difficult to know which privacy rules and regulations service providers follow, and how trustworthy they are. Currently, in Digital Health Ecosystems it is nearly impossible to control the way applications share PHI with other applications and systems. The user (a person or patient) of health-focused e-services has to make a decision on using them without reasonable and reliable information of service provider’s and their information systems’ privacy features, trust level as well as ethical principles and values [7]. Furthermore, in health care, widely used privacy concepts such as privacy as a combination of security and transparency-and-choice have failed and belief-based institutional trust will not work in the dynamic, unsecure, distributed, multivendor and multi-stakeholder environment of the Digital Health Ecosystem. Furthermore, service providers’ privacy promises are often presented to the user as “take it or leave it” manifesto without any possibility to negotiate on it. [1,8,9]. This is a doubtlessly unsatisfactory situation.

## 2. Objectives

This paper is an extension of the work originally presented to the pHealth 2019 Conference [2]. The authors’ hypothesis and requirement is that health information systems collecting, processing and sharing PHI should be ethically acceptable, trustworthy and maintain information privacy. They should also allow the service user (patient of person) to make informed decisions regarding how much the user trusts in a service provider and to what extent she/he is willing to disclose PHI. The authors state that this cannot be realized just by implementing professional ethical codes and information technology (IT) solutions such access control mechanisms, e-consent Cloud service and Blockchain technologies [10,11,12,13,14]. Instead, a novel combination of ethics, privacy, and trust is needed. On this basis, the goal of this study is to develop a proposal for a combination of ethical privacy and trust approaches that enables the building of ethically acceptable and trustworthy DHIS where the person or patient can set and enforce personal and context-aware privacy policies. Furthermore, impacts of the authors’ proposal to heath information systems and to stakeholders of the Digital Health Ecosystem are discussed.

## 3. Methods

In this paper, DHIS are understood as holistic socio-technical systems. Systems theory and systems engineering approaches according to the ISO Interoperability and Integration Reference Architecture [3,4] in combination with heuristic analysis are used to achieve the intended results. More details are discussed in Section 4.3. Existing research published in journals, conference proceedings, and standards documents has been analyzed. Features and weaknesses of widely used ethical approaches as well as privacy and trust models are studied at a fundamental level. Based on the analysis of ethical, privacy and trust models, a multidimensional proposal for DHIS with the ability to act ethically, be trustworthy and enable personal privacy rules is formulated. In that context, also principles and outcomes of the qualitative heuristics methodology are deployed.

## 4. Results

Ethics, privacy and trust are vague culture-dependent concepts without globally accepted definitions [15]. Ethics is a philosophical concept. Privacy and trust are dynamic and context-depending concepts with many meanings. From a service user’s (person, patient or consumer) point of view, they can be understood as psychological state, but have also ethical dimensions [16,17]. The way privacy, trust and ethics are implemented in information systems impacts not only how PHI is collected, processed and shared, but also how societies function. Therefore, it is necessary to analyze and understand features and problems of different ethical, trust and privacy models deployed. On this basis, the authors propose a solution that combines ethical principles, privacy and trust models in new way to support the creation of ethically acceptable, trustworthy and privacy enabling DHIS.

### 4.1. Ethics, Privacy and Trust for DHIS—Models and Problems

In daily language, ethics and morality are often used synonymously. Morality concerns the principles of right and wrong behavior. Moral values in society serve as a basis for how we understand “good” and “bad”, while ethics tries to answer the question of what actions are right or wrong in particular circumstances [18]. Ethics studies principles of ethical behavior, and the nature of ethical values. Ethical models are often divided into descriptive ethics, normative ethics and applied ethics. Descriptive ethics addresses people’s beliefs about morality and values. Normative ethics is developing standards for right and wrong behavior. It is often presented in the form of principles (e.g., non-maleficence, beneficence, autonomy, integrity and justice), and values such as fairness, accountability, responsibility, reliability, integrity and honesty. Applied ethics targets moral problems, practices and policies in professional contexts. Frequently, it is a combination of Kant’s Duty ethics and utilitarianism. Information ethics is a subdomain of applied ethics that addresses the uses and abuses of information, information technology, information systems and the use of information in decision-making [19]. It creates ethical standards and rules for processing, storing and sharing information. Computer ethics creates moral principles and rules which regulate the use of computers. For information systems, ethical questions such as an information system’s compatibility with stakeholders’ ethical values and their fairness are studied [18]. Ethical codes developed by professional organizations are typically built on ethical principles and values, e.g., the ACM Ethical Code for software Engineers and IMIA’s Ethical Code for Health Information professionals [20,21]. Table 1 summarizes goals and problems of widely used ethical models [18,19,20,21,22,23].

The most common approaches for business ethics are stockholder, stakeholder, and social contract theories. The starting point of stockholder theory is that managers are obligated to pursue profit by all legal means [27]. The stakeholder ethics requests managing business to benefit all stakeholders. Social contract theory argues that business has an ethical responsibility to enhance the welfare of society.

From viewpoint of DHIS, ethical models discussed in Table 1 have meaningful problems. Ethical principles and values are not global, and developers and users of information systems have often different opinions of them. Ethical principles, values and rules are typically presented in the form of narrative text that makes it difficult for computers and algorithms to understand them and therefore to function ethically. Business ethics can be in conflict with patients or person’s ethical needs and values (e.g., how PHI is collected and used). Principles and values of business ethics can be beneficial for a service provider’s business, but at the same time they can cause harm to the data subject (DS), and lead to the loss of information privacy. Furthermore, in today’s digital environment there is no guarantee that information collectors, service providers and secondary users keep their ethical promises, and that computer algorithms used function ethically.

Privacy is another multidimensional concept. At a general level it addresses the question “what would we like others to know of us?” It is a philosophical, psychological, sociological, and legal concept [15,17]. In a Digital Health Ecosystem, privacy exists between any actors such as persons, organizations, systems, devices, applications, processes, and even single components or objects [28]. Despite the conception that privacy is a human and constitutional right, many governments, organizations and service providers frequently interpret privacy as relative and think that the offered privacy level can be balanced with other interests such as business gain or national security [29]. Privacy is also an element in many professional codes such the aforementioned IMIA Code of Ethics for Health Information Professionals [20,21]. It has also a regulatory dimension. In the regulatory domain, two basic approaches exist: the regulatory model where the government defines privacy protection rules and the self-regulation model where industry defines privacy rules [30]. Furthermore, in real life, privacy is often understood as confidentiality, and it is implemented using security controls.

In today’s Digital Health Ecosystems, the collection and use of personal information is increasingly considered by industry as “new oil”, and by governments as a necessary tool to maintain national security and safety, and to control citizens’ behaviors. The impact of the misuse of PHI (privacy violation) is usually measured in terms of economical (monetary) harm. This causes problems in real life situations where misuse is invisible, and engendered social and psychological harm is difficult to measure in terms of money. Table 2 summarizes main features and weaknesses of commonly used privacy models [30,31,32].

Each of the privacy models presented in Table 2 has its own weaknesses in Digital Health Ecosystems, where boundaries are virtual, and PHI is dynamically and often invisibly collected and used. In many cases, a service provider’s business model is hidden and privacy rules (polices) are defined without taking into account DS’s privacy needs. Furthermore, stakeholders’ privacy features are seldom known and published. Instead, a service provider typically publishes a narrative privacy policy document made by lawyers and expects a service is either used as is or declined (take-it-or-leave-it model). As a person in real life has limited or no power to negotiate with the service provider, he or she is forced to accept service provider’s rules (policy) without sufficient and reliable information [31,33]. Consequently, the service user cannot control how PHI is used. Poor design and implementation of information systems can also generate privacy problems and increase the possibility of misusing PHI. In many today’s DHIS for example, privacy is simply managed by security tools such as authentication, access control and consent notice [8]. However, this is an insufficient solution [34].

Caused by the nature of privacy, it is difficult or even impossible to measure it directly, so proxies should be used instead. One proxy approach is the concept of risk. The level of risk is typically presented as likelihood, expected impact or score. Risk to privacy exists in all information systems, and a risk-based approach to privacy gains increasing popularity. This approach is also imbedded in the European Union General Data Protection Regulation (EU-GDPR) (article 24) that highlights the necessity to take into account risks in information systems processing PHI [42]. Typically, there is a lack of reliable information on stakeholders’ technical and organizational privacy features. The nature of Digital Health Ecosystem in general make measuring and estimating the level of privacy risk difficult or even impossible for the DS or patient. Furthermore, there are no empirical methods for a DS to determine which PHI in a situation is at-risk, what is the level of risk in a specific context and the likelihood of harm [43]. This has led to the increasing use of the concept of perceived privacy risk that can be estimated for example by surveys, use-cases and expert evaluations. Unfortunately, privacy risk perception is only a belief.

Patients and persons using services of DHIS often want to control how and by whom their PHI is used and disclosed. One solution to this problem is the deployment of multiple, formal and therefore computer understandable (and therefore harmonized) policies as defined by ISO 22,600 [44,45,46]. Unfortunately, only a few service providers in Digital Health Ecosystems support personal polices, and current laws do not force them to accept user’s privacy policies [47].

In real life, it is natural for humans to evaluate and present privacy level by using qualitative linguistic variables, i.e., words or sentences such as very strong, strong, average, low, and very low instead of crisp numbers. As mentioned in Table 2, privacy is also a fuzzy concept, and fuzzy logic can be used to measure the level of privacy in information systems. Fuzzy logic introduced by E. Zadeh (1965) is a kind of multi-value logic. It focuses on uncertainty, and it uses the approach of degrees of truth instead of traditional true/false. In fuzzy logic, a fuzzy set is group of variables having a degree of membership. Membership functions allow quantifying values of linguistic variables with words. In Fuzzy trust calculation, membership functions and IF-THEN-ELSE relationships are applied to produce the output in the form of a privacy value [48].

Benefits of fuzzy logic include the capability to quantify vague concepts (such as privacy and trust), which are difficult to present in quantitative form. Furthermore, it mimics the logic of human decision-making and presents logical rules in natural language. Fuzzy logic is strong in situations where traditional logic is insufficient such as in multi-criteria decision-making [49]. Fuzzy logic has been used for example in database protection [50], for fuzzy consent [51], for measuring privacy in online networks [52] and for privacy preserving in big data mining [53].

Similarly to privacy, trust is a contextual and dynamic concept with many definitions. Diego Gambetta provides a widely used definition: trust is a particular level of the subjective probability with which an agent assess that another agent or group will perform an action before it can be monitored or independently of capacity to ever monitor it [54]. There are many other approaches such as: trust is a psychological construct and individual feature, an institutional phenomena, expectation or acceptance of and exposure to vulnerability [55]. In real life, trust created by a human is often based on perceptions (beliefs, attitudes) of trustors’ expected features and behaviors. In real life, trust is often a multi-dimensional concept of beliefs (e.g., service provider is honest and does not misuse my PHI and the communication network is secure), positive expectations (e.g., the offered health services positively impact my health) and negative probabilities (e.g., the service will send a virus to my computer). Table 3 summarizes main features and weaknesses of widely used trust models [54,55,56,57,58,59].

Dispositional trust (also called “basic trust”) is a general tendency to trust others. It is part of personality. According to McKnight, institutional trust refers to an individual’s perceptions of an institution’s technical and organizational attributes. For example, trust in web is a combination of dispositional trust, perception of features of the internet and perceptions of vendor specific features [60]. Recommended trust uses others’ opinions (e.g., good or bad service) which are typically collected by questionnaires. Recommended trust value is produced using mathematical methods such as beta-probability [59]. Reputation is a concept commonly used in e-commerce instead of trust. It is based on general opinions and past behavior of others typically focused to quality and cost of services.

The computational trust model uses mathematical formula to calculate a trust value using direct measurements, observed features or experiences. Mathematical methods such as Bayesian probability, beta-probability, game theory, maximum likelihood, weighted average, and fuzzy logic have been used for calculation of trust level. Here, the authors have proposed attributes such as ability, willingness, transparency, predictability and reliability of service provider’s promises and service provider’s contextual features and regulatory compliancy [67]. Trust can be also created using credentials, certificates and service level agreements. In e-commerce, trust (and privacy) information is often used by a customer in decision-making, i.e., in the selection of a service provider and in the decision to use or not to use services, as well as to defining to what extent he or she has a willingness to disclose PHI.

Similarly to privacy, fuzzy logic is increasingly used in trust calculation [68,69,70]. Examples for using fuzzy logic include decision-making [71,72], e-commerce [73], multi-criteria trust management [74], and building trust in ubiquitous health care [75].

The authors state that because both trusting belief and recommended trust are based on beliefs, and dispositional trust is a personal feature, they all are unreliable in Digital Health Ecosystems. Instead, organizational trust that uses measured features of the service provider can be useful for the service user. Compared to other computational trust solutions, fuzzy trust seems to have an advantage especially because it mimics the logic of human decision-making. A common challenge for all computational trust models is the selection of measurable trust attributes and the lack of reliable information the calculation requires. This especially holds because there is currently no regulatory pressure to force service providers to publish their trust features [67].

### 4.2. A Solution for Ethically Acceptable, Privacy Preserving and Trustworthy DHIS

As stated earlier by the authors, Digital Health Ecosystems should act ethically, be trustworthy, have the ability to maintain information privacy, and support user’s personal privacy policies. For realizing this, it is necessary to take into account that DHIS is a combination of hardware and software technology such as sensors, monitoring devices, platforms, communication networks, operation systems, software applications and algorithms. According to Lederer et al. [76], privacy in ubiquitous computing environment (e.g., Digital Health Ecosystem) depends on attributes such as laws, markets, norms, ICT-architecture, information sensitivity, and perceived trust. Furthermore, trustworthiness depends on service provider’s business and ethical models, privacy level and trust features. Principles and methods used in system design and implementation are also meaningful. Furthermore, the ethical model used by the service provider together with its values and rules, as well used privacy principles and trust features should be acceptable for all stakeholders of the ecosystem, and they should also meet requirements set by laws. All this indicates that a combination of ethics, privacy and trust is inevitable.

Table 1, Table 2 and Table 3 show that all ethical, privacy and trust approaches analyzed have their own specific weaknesses, i.e., a single solution cannot meet all sets of requirements. Taking into account that the goal of health care and health service systems is to make good to patients/persons, and to avoid harm, a combination of consequentialism, computer ethics, medical professional ethics and utilitarianism is proposed by the authors for the ethical model used in DHIS. Consequentialism should be used in the form of considering consequences of services to person’s health, and of PHI collection to information privacy. Utilitarianism means that PHI should be available to Public Health, medical research and scientific research for best improving the population’s health status. For ethical values, the authors propose privacy, autonomy, usability, trust and co-operation [76], but also security, safety and value orientation [77].

According to the authors, it is necessary that the DS or a patient has not only a right but also the power to make decisions concerning the collection, use and disclosure of PHI. She or he should also have the possibility to balance the perceived harm against expected health benefits [67,78,79]. To make this true, the authors propose a privacy model where a person has a unique Intellectual Property Right concerning the collection, use and sharing of [80,81]. This new kind of right is necessary, because current privacy solutions and laws do not give a person sufficient power in Digital Health Ecosystems and in the internet to choose who, how and for what purposes his or her PHI can be collected and used. The right is also needed to prevent the increasing commodification of PHI. The proposed right cannot be a general or absolute property, and it should be balanced against information needs raised for health care, public health and scientific research.

The proposed use of Intellectual Property Right requires that the DS or patient can dynamically calculate service a provider’s or an ecosystem’s privacy level and trustworthiness before starting to use services and disclose PHI. The authors recommend that privacy level is calculated using linguistic values and fuzzy logic [67]. Service user’s privacy needs can be expressed with the help of dynamic and computer understandable policies.

As privacy and trust are interconnected in such a way that a higher level of trust indicates a lower need for privacy, trust is the third crucial element in the authors’ proposal. As discussed in Section 4.1, commonly used trust models such as dispositional trust and belief-based trust models are unreliable in Digital Health Ecosystems. Taking this and benefits of computational fuzzy trust into account, the use of computational fuzzy trust is proposed.

The calculation of trust and privacy levels is not possible without availability of sufficient and reliable information. The author’s proposal requires that all service providers and stakeholders in the Digital Health Ecosystem (e.g., health care service providers, non-regulated health service providers, DHISs, tele-operators, platform managers) publish their privacy and trust features in the form of standardized and measurable attributes. New regulations and laws are needed to force them to support transparency and publish required information. This enables the calculation of service provider specific privacy and trust levels, the formulation of remaining privacy needs and the expression of these needs in the form of an ontology-based policy model that supports computer-understandable policies [46,47,82].

Implementation of a proposed solution requires validated methods and tools. The ethical approach proposed can be implemented using methods such as ethical design, user centric, participatory and value-sensitive design in such a way that they support end-users’ behavioral expectations. For establishing privacy, methods such as Privacy-by-Design or Privacy-by-Default can be exploited. Additionally, privacy enhancing methods such as encryption, differential privacy and k-anonymity should be used for communication privacy and to guarantee information privacy in clouds and on platforms.

### 4.3. A System-Oriented, Architecture-Centric, Ontology-Based, Policy-Driven Approach to Ethically Acceptable, Privacy Preserving and Trustworthy Digital Health Ecosystems

**Figure 1:**
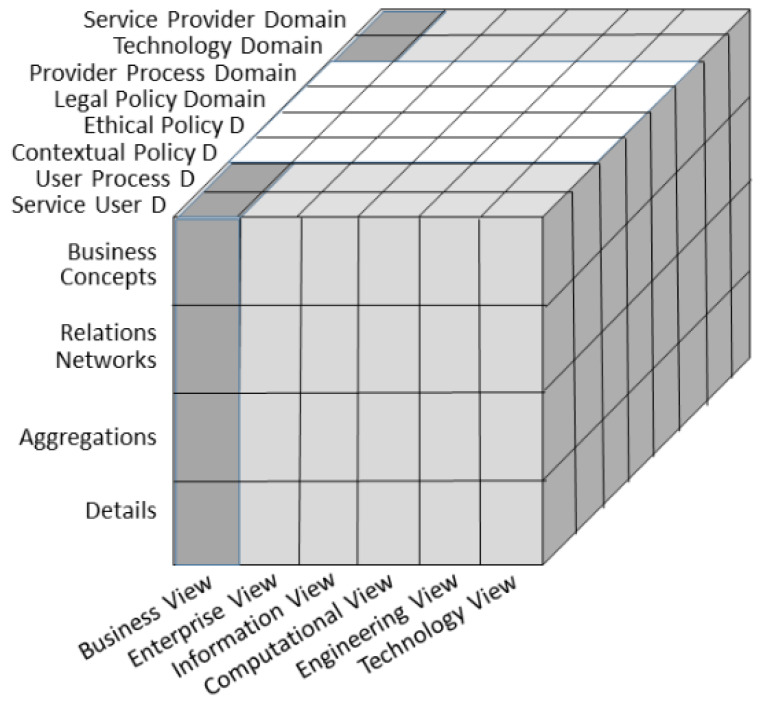
Representation of an ethically acceptable, privacy preserving and trustworthy Digital Health Ecosystems accredited by ISO 23,903 (after [77]).

System design, development and implementation including integration and interoperability challenges of the majority of current DHIS still focus on the data and information level as the proposed solution for ethically acceptable, privacy preserving and trustworthy DHIS. However, we cannot decide on the correctness and consistency of elements and their relations at that level, but need to consider context and underlying concepts of the system and its environment. Instead of representing the aforementioned principles and concepts just through data (parameters, attributes, values), they should be formally and explicitly represented through related ontologies, i.e., the involved knowledge spaces and their dependencies. The move to highly dynamic, increasingly complex, intelligent, multi-domain Digital Health Ecosystems intensifies the pressure to advance system design, management and deployment to the concept/knowledge level [3]. For meeting this challenge, a formal and consistent representation of the system and its components, their functions and relations, deploying methodologies and terminologies of the experts from all domains contributing to the ecosystem, is necessary. The Interoperability and Integration Reference Architecture approach developed by the second author and currently standardized as ISO 23,903, transforms the representation of the universe through Universal Type Theory and universal logics into a system’s architecture including a system’s development process known from systems theory and systems engineering [83]. The architectural representation of the system is instantiated using the contributing domains’ ontologies and can be managed and harmonized by top-level ontologies according to ISO/IEC 21,838 [84]. The behavior of systems and their components is defined and controlled by related policies such as security policies, privacy policies and ethical policies [4]. Figure 1 demonstrates a representation instance of an ethically acceptable, privacy preserving and trustworthy Digital Health Ecosystems accredited by ISO 23,903.

**Figure 2:**
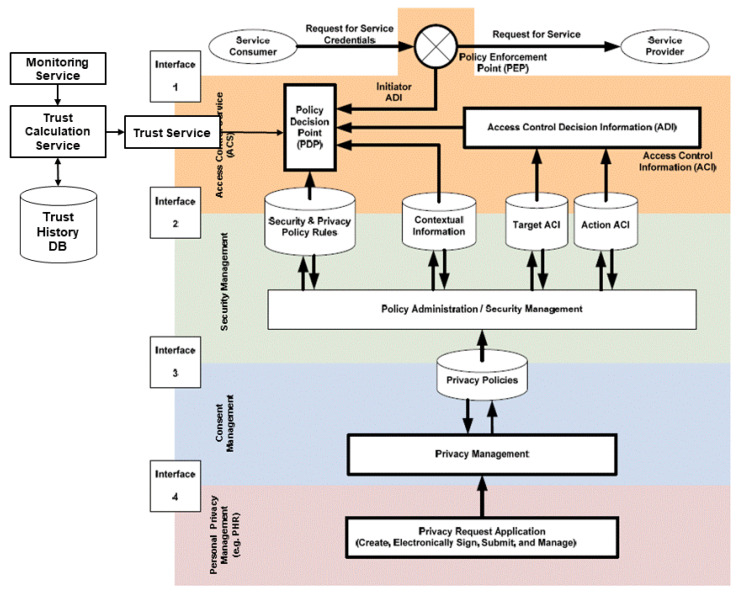
Adapted HL7/OMG Authorization Reference Model (after [4]).

A resulting implementation example for trustworthy and policy-driven access control based on the HL7/OMG PASS specification [85], extended by a trust service, is shown in Figure 2.

## 5. Discussion

The goal of this research was to develop principles for ethically acceptable and trustworthy DHIS that, from sensors and applications to system level functionality, can be designed, implemented and used in such a way that it functions ethically, is trusted, guarantees DS’s information privacy, supports user’s privacy needs and is regulatory compliant. To reach this goal, the authors have studied goals, features of common ethical, privacy and trust models and analyzed their weaknesses in Digital Health Ecosystems. Based on results obtained, the authors proposed a holistic solution for an ethically acceptable, privacy preserving and trustworthy Digital Health Ecosystem. The solution is a combination of ethical features, privacy as specific Intellectual Property Right and computational fuzzy trust. The recommended ethical solution is a combination of consequentialism, computer ethics, medical professional ethics and utilitarianism. For privacy management, calculated privacy using linguistics variables and fuzzy mathematics as well as personal context-aware privacy policies are proposed. The level of trust in the ecosystem should be calculated using stakeholders’ measured features and fuzzy mathematics. Trust and privacy calculation enables the health service user to estimate realistic privacy and trust levels of DHIS. Based on this information the user can then make a rational decision about how to use which services, and to what extent she or he has a willingness to disclose PHI.

The authors’ proposal shows a promising way towards ethically acceptable, trusted and privacy-enabled DHIS. For design and implementation of proposed solution tools such as ethical design, ethical evaluation, Privacy-by-Design and user-centric design methods can be used. In the future, it will be possible to measure the privacy level and regulatory compliance of the DHIS using algorithms, and to predict trust with the help of machine learning. For enabling the authors’ solution new regulations and laws establishing privacy as intellectual property are needed. It is also necessary to force (by law) health service providers and other stakeholders in Digital Health Information Ecosystem to publish their privacy and trust features, and in such a way, enable the service user to set their own context-aware personal privacy policies. Regulations should also define meaningful sanctions for those service providers and companying stakeholders who do not act ethically, misuse PHI and do not support transparency.

A methodological weakness in this study is that only qualitative heuristics expert methods have been used for selecting principles, values and tools proposed. For more detailed results, the deployment of quantitative methods is inevitable. Another weakness is that impacts of proposed privacy model to patients’ and DS’s willingness to disclose their PHI for scientific research and public health purposes is not studied. The acceptance and implementation of the authors’ solution require changes in information systems processing PHI. Therefore, the economic impacts on information system and health application vendors should be studied. A limitation in this paper is that ethical, privacy and trust models are studied only at fundamental level and no technology impact assessment (TIA) is made. The authors’ opinion is that this kind of study is out of the scope of this paper. There are also meaningful challenges. Compared to the current situation, the authors’ proposals require a political paradigm change, and it is unlikely that todays’ health service providers and organizations that collect PHI for secondary use will voluntarily shut down the way they are collecting and using PHI, which increasingly disregards ethical values and user’s privacy needs. Similarly, it is unlikely that governments have an automatic willingness to limit the collection and use PHI they expect to need for public purposes and citizens’ behavioral control. As mentioned before, in this paper privacy is understood as Informational Property. However, this perception might change in the future.

New service models such as digital health, precision medicine and a better understanding of the causes of disease require huge amount of multidimensional health related information. This raises the pressure to understand privacy as a public good or a commodity [17]. It might also be possible in the next decades that privacy is not considered anymore as a personal right, but understood as a statistical risk [43,86], probability [87] or commodity. Furthermore, there is at least a tendency towards understanding the sharing of PHI as societal obligation. The authors see this kind of development as undesirable for humans and for the democratic society, and the authors proposal shows a different way to approach the issue.

Considering the future work, the authors will analyze further different digital privacy and trust approaches and solutions, and search in real life measurable attributes for calculation. Based on results from a prototype solution for fuzzy logic-based privacy and trust calculation solutions, a focus on Digital Health Ecosystems will be developed. The ongoing enhancement to knowledge level approaches as presented with the system-oriented, architecture-centric, ontology-based, policy-driven interoperability and integration reference architecture model and framework will continue.

## 6. Conclusions

The success of Digital Health Ecosystem is based on the ability of its information systems and algorithms to collect, process and share PHI. Currently, the data subject has limited or no possibilities to impact what PHI is collected and shared and by whom. Furthermore, the DSs have no power to force a service provider or the government to act ethically, support privacy and to be trustworthy. Currently, privacy violations are increasingly a common practice. To change this unsatisfactory situation, the authors have proposed a new combination of ethical principles, privacy as a unique informational property right and a computational fuzzy trust approach that, in combination with regulatory support and careful implementation, can enable the building of ethically acceptable, trusted and privacy-enabled DHISs and ecosystems. If we do not understand and cannot properly represent ecosystems, we cannot control them. Therefore, a move from a data and information level to a knowledge level approach is inevitable. The system-oriented, architecture-centric, ontology-based, policy-driven interoperability and integration reference architecture model and framework paves the way for the future.

## Figures and Tables

**Table 1:** Widely used ethical models and their weaknesses.

	Ethical Model	Focus/Goals	Problems
Normative ethics	Consequentialism [24]	Focus is consequences of actions. Choices that bring more value are morally to make.	Difficult to know or calculate consequences of acts in advance.
	Utilitarianism [25]	The morally right action is that produces most overall good or wellbeing (e.g., happiness, welfare) and minimizes overall harm.	It is difficult to measure and compare impacts of acts to happiness or harm.
	Deontology [24]	Choices cannot be justified by their effects. Action is good based on its characteristics. Action should follow moral rules and laws. Duty is highest value.	Ignores consequences of actions.
	Virtue ethics [26]	Virtue (e.g., honesty, attitude) requires wisdom. Virtue and character straits of a person enable us carry out moral actions.	Based on personal characters. There is no agreement on what the virtues are. People are not honest.
Applied ethics	Computer ethics [19]	Impacts of information technology upon human values and formulation of policies for ethical use of information systems.	Impacts are difficult to measure. High-level principles are offered.
	Information ethics [18]	Ethical and moral issues arise from the development and use of information and information technologies.	Rules and principles are difficult to implement in information and communication technology (ICT) environments.
	Professional ethics [20,21,22]	Personal and corporate standards of behavior expected by professionals.	Standards are not global. High-level principles.
	Business ethics [27]	Moral principles that guide the way a business behaves.	Real principles seldom known by the customer, stockholder model is dominating.

**Table 2:** Common privacy models and their problems.

Privacy Model	Features	Weaknesses in Digital Health Ecosystem
Westin and Altman models [31,32,33,35]	Protection by limiting access of other to themselves. Selective control of access to self.	Health data is collected and used invisibly. Personal control is nearly impossible.
Communication privacy management theory (Petronio) [30,31]	Privacy has boundaries. Regulation of the degree of boundary permeability using rules.	There are no boundaries in a Digital Health Ecosystem
Privacy as contextual integrity [8,36]	Context (e.g., health care the Internet) have own principles and norms regulating information flow inside and between contexts.	Contexts are dynamic and virtual. Different contexts lead to different privacy solutions. Stronger parties can defines own norms and controls for information flow.
Online privacy [37,38]	Continuous protection of personal information in online activities.	Stakeholders’ privacy features often unknown or unreliable. Privacy approach based on social norms and laws are ineffective.
Privacy as social issue [31,32]	Privacy is a social value. Personal privacy need balanced with public, organizational and business interest.	Governmental and industrial needs often dismiss personal needs for privacy.
Privacy as Fuzzy concept [39,40,41]	A human approach to privacy using fuzzy methods mathematics.	Difficult to collect reliable input data. Output of some methods is crisp.

**Table 3:** Widely used trust models and weaknesses.

Model	Feature	Weaknesses in Digital Health Ecosystem
Disposition to trust [60,61]	General willingness to depend on others characteristics.	General personal tendency to trust is unreliable.
Organizational (institutional) trust [60,62]	Confidence that organization has promised trust features will perform beneficial actions.	Trust features are seldom known or measures, but based on beliefs in implementations.
Recommended trust [63,64]	Based on beliefs in others recommendations.	Recommendations are typically based on quality/cost and not on information privacy.
Trusting belief [60]	Subjective belief that a trustee has beneficial features.	Belief cannot be used as the base of decision.
Fuzzy approach to trust [65]	Qualitative approach to trust using natural language. Trust value is computed using fuzzy rules.	Collection of input data can be demanding. Some methods require crisp input. Determination of Fuzzy rules requires expertise.
Computational trust [66]	Mathematical methods are used to calculate trust value/rank from attributes.	Attributes are difficult to measure and seldom available.
